# Supplementary figures and images for: Brain-state invariant thalamo-cortical coordination revealed by non-linear encoders
Source: PLoS Comput Biol. 2018 Mar 22;14(3):e1006041. doi: 10.1371/journal.pcbi.1006041 (PMC5882158; doi:10.1371/journal.pcbi.1006041)

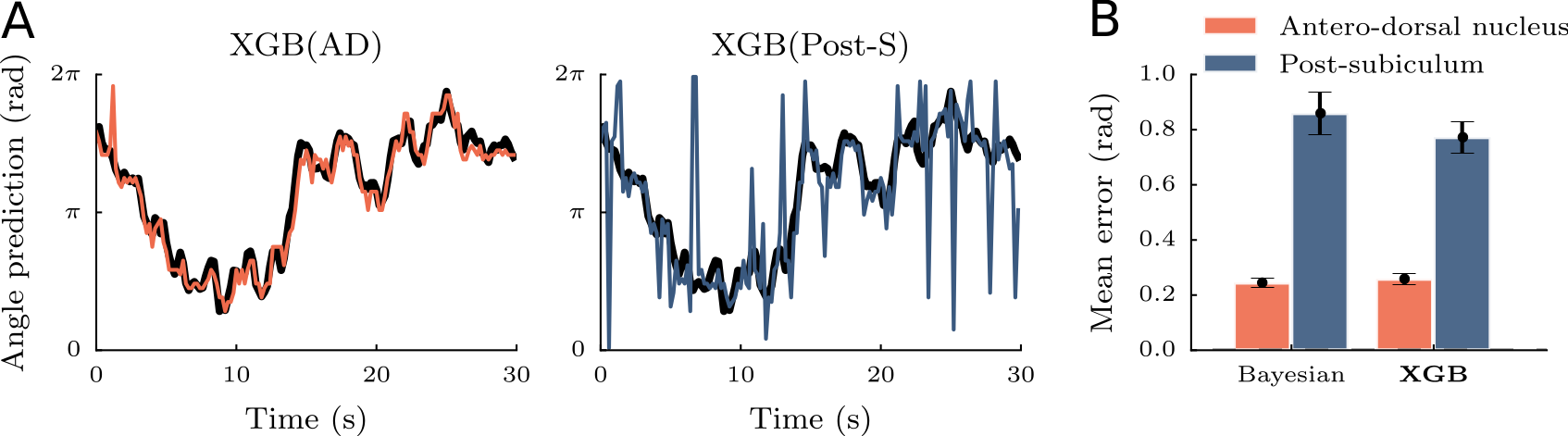

Supplement: S1 Fig — A Example of decoding for XGB during 30 seconds of head rotation for both ADn and PoSub spiking activities. The black line shows the real angular HD. B For sessions with large groups of neurons (n ≥ 7) in ADn and PoSub, the HD of the animal was decoded based on spiking activity with the classical Bayesian decoding and gradient boosted trees (XGB) over 60 angular bins. (TIF) [file pcbi.1006041.s001.tif]

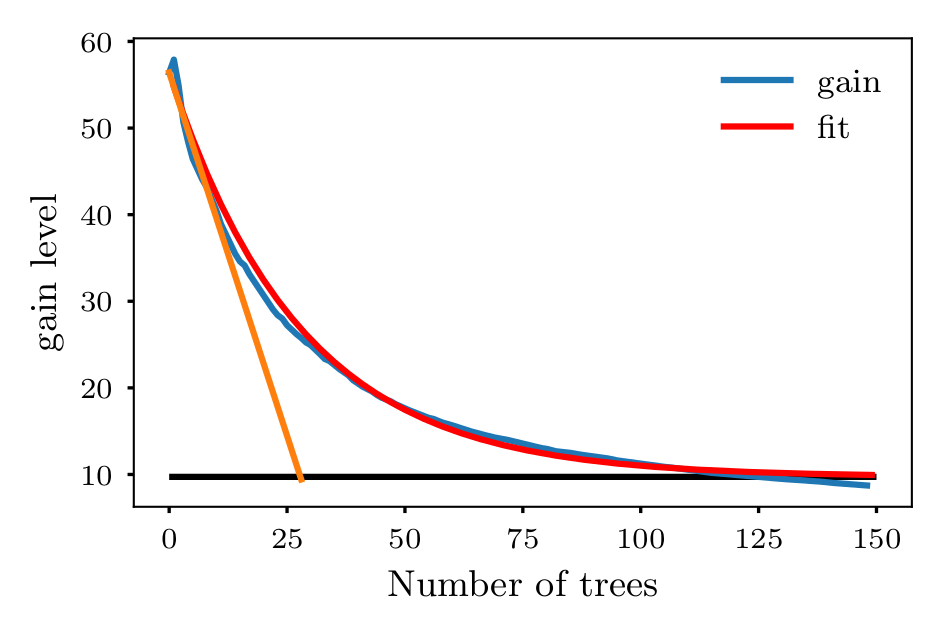

Supplement: S2 Fig — This decay was well captured by an exponential fit (red line), from which an optimal number of trees of approximatively 30 trees is derived (intersect of the linear fit at origin with the x-axis). At this stage the mean gain per tree is approximately 13 of its initial value and most of the learning has already occurred. (TIF) [file pcbi.1006041.s002.tif]

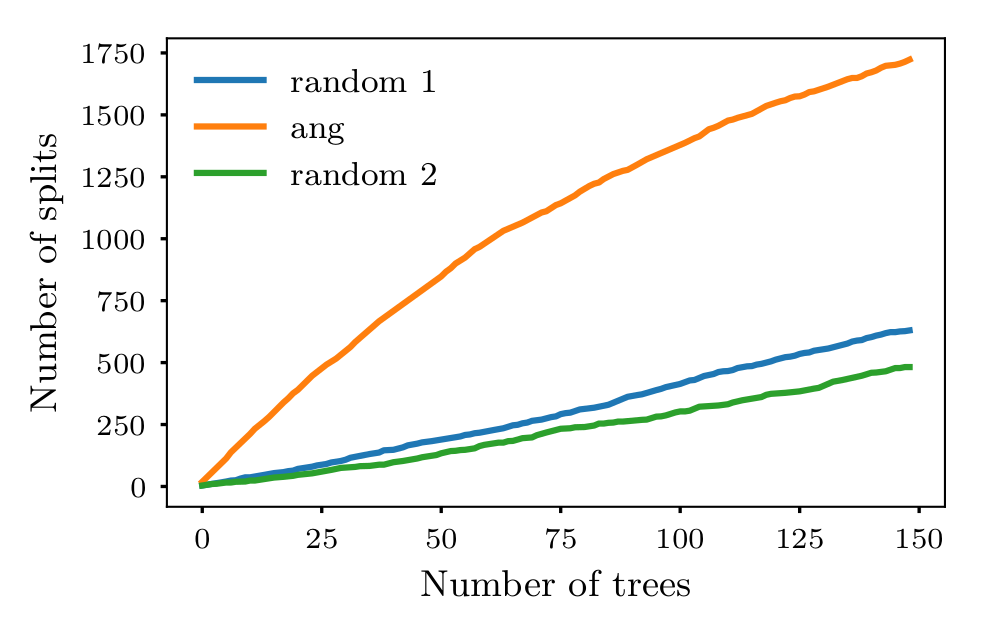

Supplement: S3 Fig — The graph illustrates the evolution of split density when learning the spike train of a HD neuron as a function of the number of trees for three features: the actual HD and two random vectors. Split density increased linearly and similarly with the number of trees in the asymptotic regime for all features. However, the increase was much higher for the HD at low tree numbers, a difference well captured by gain analysis. Note that, as the order of features in the algorithm may impact which are split first, we showed how the feature data were organized (random 1, angle and random 2). (TIF) [file pcbi.1006041.s003.tif]

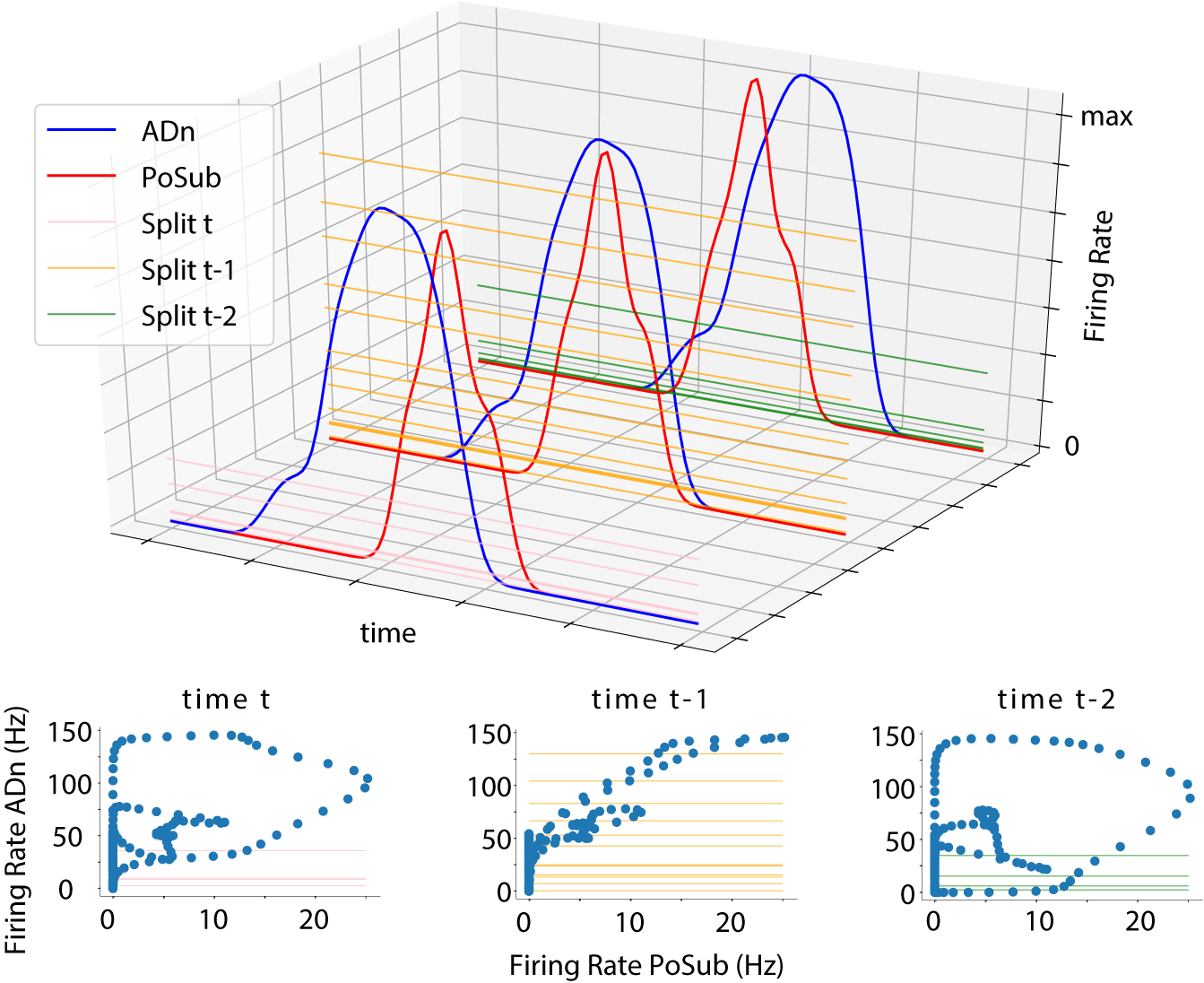

Supplement: S4 Fig — Feature space is composed of multiple copies of the activity of the feature neuron (in this case, in the ADn) at various time-lags (blue curves) to learn the target spike train (PoSub, red curves). The relationship between the two spike trains shows maximal dependence at t-1, resulting in a high number of splits by the algorithm (yellow horizontal lines). Splitting was less effective for more independent firing at t and t-2. In this example, the relationship at t-1 is trivial (linear and positively correlated). However, the quantification of these interactions give comparable values for a large variety of interactions (e.g. positive, negative or monotonically non linear). (TIF) [file pcbi.1006041.s004.tif]
